# Natural and Synthetic Sialylated Glycan Microarrays and Their Applications

**DOI:** 10.3389/fmolb.2019.00088

**Published:** 2019-09-13

**Authors:** Alyssa M. McQuillan, Lauren Byrd-Leotis, Jamie Heimburg-Molinaro, Richard D. Cummings

**Affiliations:** ^1^Department of Surgery, Beth Israel Deaconess Medical Center, National Center for Functional Glycomics, Harvard Medical School, Boston, MA, United States; ^2^Department of Microbiology and Immunology, Emory University School of Medicine, Atlanta, GA, United States

**Keywords:** sialylated glycans, glycan microarrays, synthetic glycans, natural glycans, glycan binding proteins, functional glycomics, Neu5Ac, Neu5Gc

## Abstract

This focused chapter serves as a short survey of glycan microarrays that are available with sialylated glycans, including both defined and shotgun arrays, their generation, and their utility in studying differential binding interactions to sialylated compounds, highlighting N-glycolyl (Gc) modified sialylated compounds. A brief discussion of binding interactions by lectins, antibodies, and viruses, and their relevance that have been observed with sialylated glycan microarrays is presented, as well as a discussion of cross-comparisons of array platforms and efforts to centralize and standardize the glycan microarray data.

## Introduction to Glycan Microarrays as a Tool to Study Glycan Binding Interactions

Glycan microarray technology has enabled unprecedented examination of the binding interactions between glycan binding proteins (GBPs) and host carbohydrates, as discoveries are continually being made about the significance and utility of glycans in various biological interactions. The microarray tools can be broadly grouped into (a) defined arrays with known glycan structures, and (b) undefined arrays with unknown structures, which would include arrays derived from natural source material and chemical or enzymatically modified materials that may not be fully characterized. Many of the available glycan microarray platforms contain a variety of sialylated glycans, including the N-acetyl (Ac) and N-glycolyl (Gc) forms. Sialylated glycans have been especially recognized for their significant biological roles, thus creating particular interest in sialylated glycan microarrays.

## Diversity of Glycan Microarray Platforms

### Synthesis of Sialosides

The sialosides and sialic acid derivatives that populate various microarrays are generated from different sources. Chemo-enzymatic and semi-synthetic approaches have been taken for many compounds, when root glycan structures were available for further modification by sialyltransferases or chemical approaches (Blixt et al., 2004). The most extensive variety of sialosides, containing Ac and Gc forms as well as other modifications, for use on microarrays and in other applications has been derived by the laboratory of Xi Chen (Yu et al., 2005, 2006a,b, 2011; Chokhawala et al., 2008; Linman et al., 2012; Khedri et al., 2014; Li et al., 2017). This work uses novel one-pot synthesis and combinatorial methods, and is the subject of another review in this issue (Chen et al., 2019). The diversity and biological applications of large libraries of natural and non-natural sialosides and their chemical approaches were well described in previous reviews (Deng et al., 2013; Liang et al., 2015).

### Consortium for Functional Glycomics

One of the most successful and widely used glycan microarrays has been created by the Consortium for Functional Glycomics (CFG, www.functionalglycomics.org). It is a defined, synthetic glycan microarray and is the largest publicly available glycan microarray with ~600 printed glycans on the latest version, of which approximately 20 percent are sialylated (Table 1; Blixt et al., 2004). There are 14 compounds that contain a Neu5Gc, in either an O-glycan backbone or in a ganglioside structure, as well as the monosaccharide. The CFG array has been extensively utilized to identify and study the interactions of many GBPs including galectins, C-type lectins, and siglecs; immune molecules such as anti-glycan and anti-microbial antibodies and receptors; pathogenic toxins and pathogens such as HIV and parainfluenza virus hemagglutinin-neuraminidase glycoprotein (http://www.functionalglycomics.org/static/consortium/Library.shtml) (Stowell et al., 2008; Hickey et al., 2010; Alymova et al., 2012, 2016; Blackler et al., 2016; Hussein et al., 2016; Jobling, 2016; Jones et al., 2016; Malik et al., 2016; Petrova et al., 2016; Schroeder et al., 2016; Vainauskas et al., 2016; Collins et al., 2017; Noach et al., 2017). The CFG glycan microarray was instrumental in identifying the anti-carbohydrate antibodies found in human intravenous immunoglobulin (IVIG) (Schneider et al., 2015) and helping to elucidate the specificity for Neu5Gc of an *E. coli* derived subtilase toxin (Day et al., 2017).

**Table 1:** List of sialylated glycans present on the CFG glycan microarray, representing about 20% of the total number of glycans present.

**Sialylated glycans on CFG array**	
Neu5Aca-Sp8	Neu5Aca2-6(Galb1-3)GlcNAcb1-4Galb1-4Glcb-Sp10
Neu5Aca-Sp11	Neu5Aca2-6Galb1-4GlcNAcb1-2Mana1-3Manb1-4GlcNAcb1-4GlcNAc-Sp12
Neu5Acb-Sp8	Neu5Aca2-6Galb1-4GlcNAcb1-2Mana1-6(Galb1-4GlcNAcb1-2Mana1-3)Manb1-4GlcNAcb1-4GlcNAcb-Sp12
Neu5Aca2-3(6S)Galb1-4GlcNAcb-Sp8	Neu5Aca2-6Galb1-4GlcNAcb1-2Mana1-6(GlcNAcb1-2Mana1-3)Manb1-4GlcNAcb1-4GlcNAcb-Sp12
Neu5Aca2-3Galb-Sp8	Neu5Aca2-6Galb1-4GlcNAcb1-2Mana1-6(GlcNAcb1-4)(Neu5Aca2-6Galb1-4GlcNAcb1-2Mana1-3)Manb1-4GlcNAcb1-4GlcNAcb-Sp21
Neu5Aca2-3Galb1-3(6S)GalNAca-Sp8	Neu5Aca2-6Galb1-4GlcNAcb1-2Mana1-6(Mana1-3)Manb1-4GlcNAcb1-4GlcNAc-Sp12
Neu5Aca2-3Galb1-3(6S)GlcNAc-Sp8	Neu5Aca2-6Galb1-4GlcNAcb1-2Mana1-6(Neu5Aca2-6Galb1-4GlcNAcb1-2Man-a1-3)Manb1-4GlcNAcb1-4GlcNAcb-Sp21
Neu5Aca2-3Galb1-3(Fuca1-4)GlcNAcb-Sp8	Neu5Aca2-6Galb1-4GlcNAcb1-2Mana1-6(Neu5Aca2-6Galb1-4GlcNAcb1-2Mana1-3)Manb1-4GlcNAcb1-4(Fuca1-6)GlcNAcb-Sp24
Neu5Aca2-3Galb1-3(Fuca1-4)GlcNAcb1-3Galb1-3(Fuca1-4)GlcNAcb-Sp0	Neu5Aca2-6Galb1-4GlcNAcb1-2Mana1-6(Neu5Aca2-6Galb1-4GlcNAcb1-2Mana1-3)Manb1-4GlcNAcb1-4GlcNAcb-Sp12
Neu5Aca2-3Galb1-3(Fuca1-4)GlcNAcb1-3Galb1-4(Fuca1-3)GlcNAcb-Sp0	Neu5Aca2-6Galb1-4GlcNAcb1-2Mana1-6(Neu5Aca2-6Galb1-4GlcNAcb1-2Mana1-3)Manb1-4GlcNAcb1-4GlcNAcb-Sp24
Neu5Aca2-3Galb1-3GalNAca-Sp14	Neu5Aca2-6Galb1-4GlcNAcb1-2Mana1-6Manb1-4GlcNAcb1-4GlcNAc-Sp12
Neu5Aca2-3Galb1-3GalNAca-Sp8	Neu5Aca2-6Galb1-4GlcNAcb1-3Galb1-3GlcNAcb-Sp0
Neu5Aca2-3Galb1-3GalNAcb1-3Gala1-4Galb1-4Glcb-Sp0	Neu5Aca2-6Galb1-4GlcNAcb1-3Galb1-4(Fuca1-3)GlcNAcb1-3Galb1-4(Fuca1-3)GlcNAcb-Sp0
Neu5Aca2-3Galb1-3GalNAcb1-4(Neu5Aca2-3)Galb1-4Glcb-Sp0	Neu5Aca2-6Galb1-4GlcNAcb1-3Galb1-4GlcNAcb-Sp0
Neu5Aca2-3Galb1-3GalNAcb1-4Galb1-4Glcb-Sp0	Neu5Aca2-6Galb1-4GlcNAcb1-3Galb1-4GlcNAcb1-2Mana1-6(Neu5Aca2-6Galb1-4GlcNAcb1-3Galb1-4GlcNAcb1-2Mana1-3)Manb1-4GlcNAcb1-4GlcNAcb-Sp12
Neu5Aca2-3Galb1-3GlcNAcb-Sp0	Neu5Aca2-6Galb1-4GlcNAcb1-3Galb1-4GlcNAcb1-3Galb1-4GlcNAcb-Sp0
Neu5Aca2-3Galb1-3GlcNAcb1-2Mana-Sp0	Neu5Aca2-6Galb1-4GlcNAcb1-3Galb1-4GlcNAcb1-3Galb1-4GlcNAcb1-2Mana1-6(Neu5Aca2-6Galb1-4GlcNAcb1-3Galb1-4GlcNAcb1-3Galb1-4GlcNAcb1-2Mana1-3)Manb1-4GlcNAcb1-4GlcNAcb-Sp12
Neu5Aca2-3Galb1-3GlcNAcb1-2Mana1-6(GlcNAcb1-4)(Neu5Aca2-3Galb1-3GlcNAcb1-2Mana1-3)Manb1-4GlcNAcb1-4GlcNAc-Sp21	Neu5Aca2-6Galb1-4GlcNAcb1-3Galb1-4GlcNAcb1-3GalNAca-Sp14
Neu5Aca2-3Galb1-3GlcNAcb1-2Mana1-6(Neu5Aca2-3Galb1-3GlcNAcb1-2Mana1-3)Manb1-4GlcNAcb1-4GlcNAc-Sp19	Neu5Aca2-6Galb1-4GlcNAcb1-3Galb1-4GlcNAcb1-6(Galb1-3)GalNAca-Sp14
Neu5Aca2-3Galb1-3GlcNAcb1-3Galb1-3GlcNAcb-Sp0	Neu5Aca2-6Galb1-4GlcNAcb1-3Galb1-4GlcNAcb1-6(Neu5Aca2-6Galb1-4GlcNAcb1-3Galb1-4GlcNAcb1-3)GalNAca-Sp14
Neu5Aca2-3Galb1-3GlcNAcb1-3Galb1-4GlcNAcb-Sp0	Neu5Aca2-6Galb1-4GlcNAcb1-3GalNAc-Sp14
Neu5Aca2-3Galb1-3GlcNAcb1-3GalNAca-Sp14	Neu5Aca2-6Galb1-4GlcNAcb1-4Mana1-6(GlcNAcb1-4)(Neu5Aca2-6Galb1-4GlcNAcb1-4(Neu5Aca2-6Galb1-4GlcNAcb1-2)Mana1-3)Manb1-4GlcNAcb1-4GlcNAcb-Sp21
Neu5Aca2-3Galb1-3GlcNAcb1-6GalNAca-Sp14	Neu5Aca2-6Galb1-4GlcNAcb1-6(Fuca1-2Galb1-3GlcNAcb1-3)Galb1-4Glc-Sp21
Neu5Aca2-3Galb1-4(6S)GlcNAcb-Sp8	Neu5Aca2-6Galb1-4GlcNAcb1-6(Fuca1-2Galb1-4(Fuca1-3)GlcNAcb1-3)Galb1-4Glc-Sp21
Neu5Aca2-3Galb1-4(Fuca1-3)(6S)GlcNAcb-Sp8	Neu5Aca2-6Galb1-4GlcNAcb1-6(Galb1-3)GalNAca-Sp14
Neu5Aca2-3Galb1-4(Fuca1-3)GlcNAcb-Sp0	Neu5Aca2-6Galb1-4GlcNAcb1-6(Galb1-3GlcNAcb1-3)Galb1-4Glcb-Sp21
Neu5Aca2-3Galb1-4(Fuca1-3)GlcNAcb-Sp8	Neu5Aca2-6Galb1-4GlcNAcb1-6(Neu5Aca2-6Galb1-4GlcNAcb1-2)Mana1-6(GlcNAcb1-4)(Neu5Aca2-6Galb1-4GlcNAcb1-2Mana1-3)Manb1-4GlcNAcb1-4GlcNAcb-Sp21
Neu5Aca2-3Galb1-4(Fuca1-3)GlcNAcb1-2Mana-Sp0	Neu5Aca2-6Galb1-4GlcNAcb1-6(Neu5Aca2-6Galb1-4GlcNAcb1-2)Mana1-6(GlcNAcb1-4)(Neu5Aca2-6Galb1-4GlcNAcb1-4(Neu5Aca2-6Galb1-4GlcNAcb1-2)Mana1-3)Manb1-4GlcNAcb1-4GlcNAcb-Sp21
Neu5Aca2-3Galb1-4(Fuca1-3)GlcNAcb1-3Galb-Sp8	Neu5Aca2-6Galb1-4GlcNAcb1-6GalNAca-Sp14
Neu5Aca2-3Galb1-4(Fuca1-3)GlcNAcb1-3Galb1-4(Fuca1-3)GlcNAcb1-3Galb1-4(Fuca1-3)GlcNAcb-Sp0	Neu5Aca2-6GalNAca-Sp8
Neu5Aca2-3Galb1-4(Fuca1-3)GlcNAcb1-3Galb1-4GlcNAcb-Sp8	Neu5Aca2-6GalNAcb1-4(6S)GlcNAcb-Sp8
Neu5Aca2-3Galb1-4(Fuca1-3)GlcNAcb1-3GalNAca-Sp14	Neu5Aca2-6GalNAcb1-4GlcNAcb-Sp0
Neu5Aca2-3Galb1-4(Fuca1-3)GlcNAcb1-6(Galb1-3)GalNAca-Sp14	Neu5Aca2-6GlcNAcb1-4GlcNAc-Sp21
Neu5Aca2-3Galb1-4(Fuca1-3)GlcNAcb1-6(Neu5Aca2-3Galb1-3)GalNAc-Sp14	Neu5Aca2-6GlcNAcb1-4GlcNAcb1-4GlcNAc-Sp21
Neu5Aca2-3Galb1-4(Neu5Aca2-3Galb1-3)GlcNAcb-Sp8	Galb1-4GlcNAcb1-6(Neu5Aca2-6Galb1-3GlcNAcb1-3)Galb1-4Glc-Sp21
Neu5Aca2-3Galb1-4Glcb-Sp0	Galb1-4GlcNAcb1-2Mana1-6(Neu5Aca2-6Galb1-4GlcNAcb1-2Mana1-3)Manb1-4GlcNAcb1-4GlcNAcb-Sp12
Neu5Aca2-3Galb1-4Glcb-Sp8	Mana1-6(Neu5Aca2-6Galb1-4GlcNAcb1-2Mana1-3)Manb1-4GlcNAcb1-4GlcNAc-Sp12
Neu5Aca2-3Galb1-4GlcNAcb-Sp0	Neu5Acb2-6GalNAca-Sp8
Neu5Aca2-3Galb1-4GlcNAcb-Sp8	Neu5Acb2-6(Galb1-3)GalNAca-Sp8
Neu5Aca2-3Galb1-4GlcNAcb1-2Mana-Sp0	Neu5Acb2-6Galb1-4GlcNAcb-Sp8
Neu5Aca2-3Galb1-4GlcNAcb1-2Mana1-6(GlcNAcb1-4)(Neu5Aca2-3Galb1-4GlcNAcb1-2Mana1-3)Manb1-4GlcNAcb1-4GlcNAcb-Sp21	Neu5Aca2-8Neu5Aca-Sp8
Neu5Aca2-3Galb1-4GlcNAcb1-2Mana1-6(Neu5Aca2-3Galb1-4GlcNAcb1-2Mana1-3)Manb1-4GlcNAcb1-4(Fuca1-6)GlcNAcb-Sp24	Neu5Aca2-8Neu5Acb-Sp17
Neu5Aca2-3Galb1-4GlcNAcb1-2Mana1-6(Neu5Aca2-3Galb1-4GlcNAcb1-2Mana1-3)Manb1-4GlcNAcb1-4GlcNAcb-Sp12	Neu5Aca2-8Neu5Aca2-8Neu5Aca-Sp8
Neu5Aca2-3Galb1-4GlcNAcb1-3Galb-Sp8	Neu5Aca2-8Neu5Aca2-8Neu5Acb-Sp8
Neu5Aca2-3Galb1-4GlcNAcb1-3Galb1-3GlcNAcb-Sp0	Neu5Aca2-8Neu5Aca2-3Galb1-4Glcb-Sp0
Neu5Aca2-3Galb1-4GlcNAcb1-3Galb1-4(Fuca1-3)GlcNAcb-Sp0	Neu5Aca2-8Neu5Gca2-3Galb1-4GlcNAc-Sp0
Neu5Aca2-3Galb1-4GlcNAcb1-3Galb1-4GlcNAcb-Sp0	Neu5Aca2-8Neu5Aca2-3Galb1-4GlcNAc-Sp0
Neu5Aca2-3Galb1-4GlcNAcb1-3Galb1-4GlcNAcb1-2Mana1-6(Neu5Aca2-3Galb1-4GlcNAcb1-3Galb1-4GlcNAcb1-2Mana1-3)Manb1-4GlcNAcb1-4GlcNAcb-Sp12	Neu5Aca2-8Neu5Aca2-8Neu5Aca2-3Galb1-4Glcb-Sp0
Neu5Aca2-3Galb1-4GlcNAcb1-3Galb1-4GlcNAcb1-3Galb1-4GlcNAcb-Sp0	Neu5Aca2-8Neu5Aca2-3Galb1-3GalNAcb1-4(Neu5Aca2-3)Galb1-4Glc-Sp21
Neu5Aca2-3Galb1-4GlcNAcb1-3Galb1-4GlcNAcb1-3Galb1-4GlcNAcb1-2Mana1-6(Neu5Aca2-3Galb1-4GlcNAcb1-3Galb1-4GlcNAcb1-3Galb1-4GlcNAcb1-2Mana1-3)Manb1-4GlcNAcb1-4GlcNAcb-Sp12	Neu5Aca2-8Neu5Aca2-3Galb1-3GalNAcb1-4(Neu5Aca2-8Neu5Aca2-3)Galb1-4Glcb-Sp0
Neu5Aca2-3Galb1-4GlcNAcb1-3Galb1-4GlcNAcb1-3GalNAca-Sp14	GalNAcb1-4(Neu5Aca2-8Neu5Aca2-3)Galb1-4Glcb-Sp0
Neu5Aca2-3Galb1-4GlcNAcb1-3Galb1-4GlcNAcb1-6(Galb1-3)GalNAca-Sp14	GalNAcb1-4(Neu5Aca2-8Neu5Aca2-8Neu5Aca2-8Neu5Aca2-3)Galb1-4Glcb-Sp0
Neu5Aca2-3Galb1-4GlcNAcb1-3Galb1-4GlcNAcb1-6(Neu5Aca2-3Galb1-4GlcNAcb1-3Galb1-4GlcNAcb1-3)GalNAca-Sp14	GalNAcb1-4(Neu5Aca2-8Neu5Aca2-8Neu5Aca2-3)Galb1-4Glcb-Sp0
Neu5Aca2-3Galb1-4GlcNAcb1-3GalNAc-Sp14	Galb1-3GalNAcb1-4(Neu5Aca2-8Neu5Aca2-8Neu5Aca2-3)Galb1-4Glcb-Sp21
Neu5Aca2-3Galb1-4GlcNAcb1-4Mana1-6(GlcNAcb1-4)(Neu5Aca2-3Galb1-4GlcNAcb1-4(Neu5Aca2-3Galb1-4GlcNAcb1-2)Mana1-3)Manb1-4GlcNAcb1-4GlcNAcb-Sp21	Neu5Gca-Sp8
Neu5Aca2-3Galb1-4GlcNAcb1-6(Galb1-3)GalNAca-Sp14	Neu5Gca2-3Galb1-3(Fuca1-4)GlcNAcb-Sp0
Neu5Aca2-3Galb1-4GlcNAcb1-6(Neu5Aca2-3Galb1-3)GalNAca-Sp14	Neu5Gca2-3Galb1-3GlcNAcb-Sp0
Neu5Aca2-3Galb1-4GlcNAcb1-6(Neu5Aca2-3Galb1-4GlcNAcb1-2)Mana1-6(GlcNAcb1-4)(Neu5Aca2-3Galb1-4GlcNAcb1-2Mana1-3)Manb1-4GlcNAcb1-4GlcNAcb-Sp21	Neu5Gca2-3Galb1-4(Fuca1-3)GlcNAcb-Sp0
Neu5Aca2-3Galb1-4GlcNAcb1-6(Neu5Aca2-3Galb1-4GlcNAcb1-2)Mana1-6(GlcNAcb1-4)(Neu5Aca2-3Galb1-4GlcNAcb1-4(Neu5Aca2-3Galb1-4GlcNAcb1-2)Mana1-3)Manb1-4GlcNAcb1-4GlcNAcb-Sp21	Neu5Gca2-3Galb1-4GlcNAcb-Sp0
Neu5Aca2-3Galb1-4GlcNAcb1-6(Neu5Aca2-3Galb1-4GlcNAcb1-3)GalNAca-Sp14	Neu5Gca2-3Galb1-4Glcb-Sp0
Neu5Aca2-3Galb1-4GlcNAcb1-6GalNAca-Sp14	Neu5Gca2-6GalNAca-Sp0
Neu5Aca2-3GalNAca-Sp8	Neu5Gca2-6Galb1-4GlcNAcb-Sp0
Neu5Aca2-3GalNAcb1-4GlcNAcb-Sp0	Neu5Gcb2-6Galb1-4GlcNAc-Sp8
GalNAcb1-4(Neu5Aca2-3)Galb1-4GlcNAcb-Sp0	Neu5Gca2-8Neu5Gca2-3Galb1-4GlcNAc-Sp0
GalNAcb1-4(Neu5Aca2-3)Galb1-4GlcNAcb-Sp8	Neu5Gca2-8Neu5Aca2-3Galb1-4GlcNAc-Sp0
GalNAcb1-4(Neu5Aca2-3)Galb1-4GlcNAcb1-3GalNAca-Sp14	Neu5Gca2-8Neu5Gca2-3Galb1-4GlcNAcb1-3Galb1-4GlcNAc-Sp0
GlcNAcb1-6(Neu5Aca2-3Galb1-3)GalNAca-Sp14	Neu5Gca2-8Neu5Gca2-6Galb1-4GlcNAc-Sp0
Fuca1-2Galb1-3GalNAcb1-4(Neu5Aca2-3)Galb1-4Glcb-Sp0	Neu5,9Ac_2_a-Sp8
Fuca1-2Galb1-3GalNAcb1-4(Neu5Aca2-3)Galb1-4Glcb-Sp9	Neu5,9Ac2a2-6Galb1-4GlcNAcb-Sp8
GalNAcb1-4(Neu5Aca2-3)Galb1-4Glcb-Sp0	Neu5,9Ac2a2-3Galb1-4GlcNAcb-Sp0
Galb1-3GalNAcb1-4(Neu5Aca2-3)Galb1-4Glcb-Sp0	Neu5,9Ac2a2-3Galb1-3GlcNAcb-Sp0
Neu5Aca2-3Galb1-3GalNAcb1-4(Neu5Aca2-8Neu5Aca2-3)Galb1-4Glcb-Sp0	
Neu5Aca2-6(Neu5Aca2-3)GalNAca-Sp8	
Neu5Aca2-6(Neu5Aca2-3Galb1-3)GalNAca-Sp14	
Neu5Aca2-6(Neu5Aca2-3Galb1-3)GalNAca-Sp8	**Linker structures**
Neu5Aca2-3Galb1-4GlcNAcb1-2Mana1-6(Neu5Aca2-6Galb1-4GlcNAcb1-2Mana1-3)Manb1-4GlcNAcb1-4GlcNAcb-Sp12	Sp0 : -CH2CH2NH2
Neu5Aca2-6Galb1-4GlcNAcb1-2Mana1-6(Neu5Aca2-3Galb1-4GlcNAcb1-2Mana1-3)Manb1-4GlcNAcb1-4GlcNAcb-Sp12	Sp8 : -CH2CH2CH2NH2
Galb1-4(Fuca1-3)GlcNAcb1-6(Neu5Aca2-6(Neu5Aca2-3Galb1-3)GlcNAcb1-3)Galb1-4Glc-Sp21	Sp9 : -CH2CH2CH2CH2CH2NH2
Neu5Aca2-6Galb1-4 GlcNAcb1-6(Neu5Aca2-6Galb1-4GlcNAcb1-3)GalNAca-Sp14	Sp10 : -NHCOCH2NH
Neu5Aca2-6Galb-Sp8	Sp11 : -OCH2C6H4-p-NHCOCH2NH
Neu5Aca2-6Galb1-4(6S)GlcNAcb-Sp8	Sp12 : Asparagine
Neu5Aca2-6Galb1-4Glcb-Sp0	Sp14 : Threonine
Neu5Aca2-6Galb1-4Glcb-Sp8	Sp17 : -OCH2C6H4NH2
Neu5Aca2-6Galb1-4GlcNAcb-Sp0	Sp19 : EN or NK
Neu5Aca2-6Galb1-4GlcNAcb-Sp8	Sp21 : -N(CH3)-O-(CH2)2-NH2
Neu5Aca2-6Galb1-4GlcNAcb1-2Man-Sp0	Sp24 : KVANKT
Neu5Aca2-6(Galb1-3)GalNAca-Sp14	
Neu5Aca2-6(Galb1-3)GalNAca-Sp8	

*See the CFG website for more information. Spacer (linker) structures are provided in the table; a- or b- in the structure and prior to the Sp designation indicates the alpha or beta linkage; (6S) indicates sulfate groups and linkage. Yellow highlight indicates the structures that specifically contain Gc sialic acid as opposed to Ac or a derivative*.

**Figure 1:**
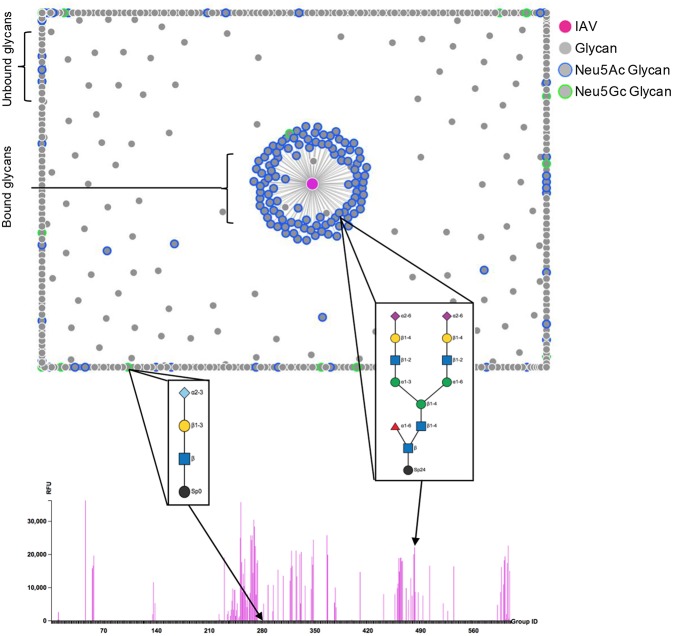
Influenza A virus binding to CFG array. The strength of IAV binding to each glycan on the CFG array is visualized by the force plot, where bound glycans are linked to the IAV spot (magenta) in the middle and unbound glycans are pushed to the periphery. Neu5Ac and Neu5Gc sialylated glycans are highlighted in blue and green, respectively. The structures corresponding to one example each of Neu5Ac and Neu5Gc terminating glycans are displayed and the binding to each structure is highlighted on the bar chart comparing RFU. Glycan cartoon key: light blue diamond- Neu5Gc, purple diamond- Neu5Ac, yellow circle- Gal, blue square- GlcNAc, green circle- Man, red triangle- Fuc, black circle- linker. Figure produced using the GLycan Array Dashboard (GLAD) program, developed by the Cummings group (Mehta and Cummings, 2019), website- https://glycotoolkit.com/Tools/GLAD/.

In addition to mapping the broad recognition of GBPs, the utility of the CFG glycan microarray lies in its versatility for multiple types of comparative binding studies. The arrays can be used to compare sample “sets,” as in wild-type vs. mutant proteins or pathogens. Additionally, assay conditions for the same sample can be compared in order to reveal environmental features of the binding interaction such as calcium dependence. While each array is considered an independent experiment, the “relative” binding feature allows for comparisons to be made, and all of the experiments, scanning, and data analysis are performed in a common location to decrease other variables. The glycans found on the array have been chemo/enzymatically produced, and often structures are replicated with one difference, for example in the terminal sialic acid linkage or even the linker molecule, allowing for fine specificity comparisons of what was once thought erroneously to be “minor” differences. As such, this collection of glycans intrinsically allows for comparative analysis of carbohydrate structural determinants that are important for recognition and binding, and it becomes important to look at what structures are *bound* as well as related structures that are *unbound*. For example, examination of the sialic acid recognition of influenza A virus (IAV) hemagglutinin has been completed using the CFG array (Bradley et al., 2011a; Gulati et al., 2013, 2014; Byrd-Leotis et al., 2014), and those interactions are exclusively limited to the Neu5Ac form as seen in multiple studies using many IAV strains, with no binding to Neu5Gc (Figure 1), as visualized with the GLycan Array Dashboard (GLAD) analytics program (Mehta and Cummings, 2019). Interestingly, as swine isolates of IAV were studied on the CFG array, the lack of Neu5Gc binding was maintained, indicating that while such glycans are able to be synthesized within the swine host, they are still unable to be recognized by influenza HA, highlighting the specificity of the receptor binding pocket for the N-acetyl over the N-glycolyl moiety (Bradley et al., 2011a). In broader strokes, the comparative nature of the CFG array has been utilized extensively as a diagnostic indicator of pandemic potential for influenza A viruses as binding preference to terminal sialic acid linkage conformations that are indicative of avian (α2,3-Sia) or human (α2,6-Sia) adaptation are easily visualized (Gulati et al., 2013, 2014; Byrd-Leotis et al., 2014). While the synthetic glycan arrays are powerful tools to elucidate the possible recognition by the pathogen or host protein under examination, the biological relevance of such interactions cannot be known. The question is 2-fold: are the glycans that are synthesized in the laboratory representative of those in the host and if so, are they present in enough abundance and localized appropriately in order to be utilized as receptors? In order to study these questions, a different process for generating the glycan microarrays was developed.

### Glycans From Natural Sources

**Figure 2:**
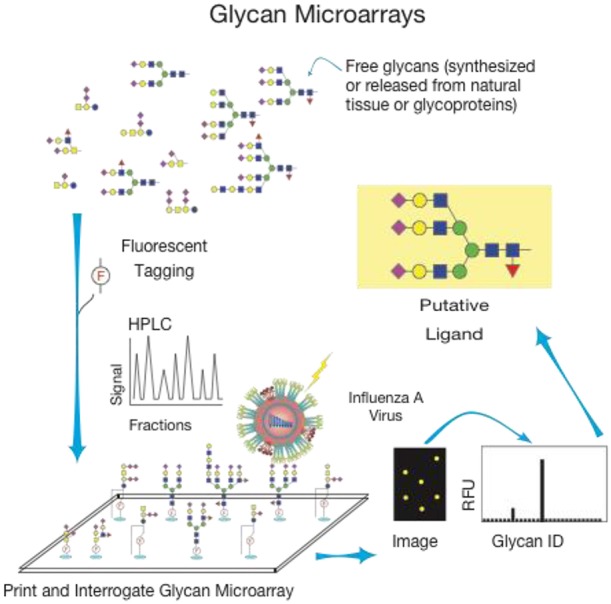
General method for creation and use of glycan microarrays from natural materials.

Natural glycan microarrays are so designated because the glycans have been directly isolated from host tissues, functionalized, typically partly purified, and then printed on glass slides to create a microarray (Figure 2; Song et al., 2011a). These arrays are direct representations of the glycan profiles, including both structural content and relative abundance, of the target host tissue and therefore provide relevant biological information about the binding interactions. These arrays are often presented as shotgun arrays, meaning that the glycans are not sequenced prior to printing and as such, the investigators are reliant on interactions with GBPs to prioritize the glycan characterization. Multiple strategies for glycan release from the tissue have been outlined including enzymatic release (Heimburg-Molinaro et al., 2011; Song et al., 2014) and chemical release using sodium hypochlorite (NaOCl or bleach) (Song et al., 2016). Once the glycans have been released, the functionalization process varies widely with respect to the linkers used, the substrate for printing, and the methods of analysis. This strategy has generated great excitement across many fields of study to look at the natural receptors and binding partners for many biologically relevant questions, including the interactions of influenza viruses with the native host receptors. Because different sialic acids can be detected by several methods, including lectin binding, the natural arrays can begin to be characterized using reagents of known specificity to help categorize and map out some of the structural features present in the tissue/cells before any detailed structural information is obtained. A natural shotgun glycan array comprised of glycans from swine lungs reveals the presence of sialylated glycans within the tissue that are potential receptors for various strains of IAV (Byrd-Leotis et al., 2014). This work showed a practical, biologically-relevant use of the “shotgun glycomics” method with pig lung tissue and allowed for studies of the natural receptors for influenza. The presence of sialylated glycans was able to be discerned clearly with the use of chromatographic separation, mass spectrometry, and the binding of plant lectins with known specificity, before looking at virus binding. Because the binding specificities of viruses chosen for study had been established on the defined CFG array, predictions regarding the types of glycans on the natural array that would be recognized by IAV could be made. The predictions held true for many structures which were present on both arrays, but IAV binding also revealed interactions with new structures that were not present on the defined array, for example triantennary α2,3 and α2,6 Sia-terminating, core fucosylated N-glycans. As such, the natural shotgun glycan microarray of the pig lung exposed biologically relevant receptors for influenza A viruses that would not have been discovered by relying on the synthetic arrays alone.

A more recent study used a human lung shotgun glycan microarray, derived from total human lung glycoproteins, which would not be expected to contain Neu5Gc. Indeed, this was observed, as this glycan microarray helped to identify both sialylated (NeuAc), and phosphorylated oligomannose-type N-glycans recognized by various strains of influenza virus (Byrd-Leotis et al., 2019). The work complemented the development of a synthetic N-glycan microarray comprised of Asn-linked oligosaccharides terminated in various branching structures and with either α2,3- or α2,6-sialylation (Gao et al., 2019). Many recent influenza virus isolates showed preferential binding to the phosphorylated oligomannose-type N-glycans compared to sialylated glycans (Byrd-Leotis et al., 2019).

### Specialized Sialo-Arrays and Tools

Many groups have generated specialized arrays for the study of specific glycan interactions. Arrays of microbial polysaccharides, which contain sialic acids as well as various derivatives such as 2-keto-3-deoxy-D-glycero-D-galacto-nononic acid (KDN), have been generated to study many types of host-pathogen interactions, for example the examination of human serum antibodies and innate and adaptive immune proteins (Wang et al., 2002). Specific N-glycan microarrays have been generated with and without terminal sialic acid, and these arrays can be tested with GBPs to look for interactions with sialylated and non-sialylated structures, but in addition can be modified by enzymatic or chemical methods to create new sialylated epitopes with Neu5Ac, Neu5Gc, and other modified sialic acids for binding studies (Hamilton et al., 2017). An array of sialic acid derivatives, not found on the CFG array or other arrays, was created using a novel method to examine binding interactions with these more unusual sialylated compounds (Bradley et al., 2011b; Song et al., 2011b). The array was informative for lectin binding, as well as influenza and parainfluenza binding studies. While most influenza strains maintained a lack of binding to NeuGc derivatives, a new binding partner was seen- a lactoyl Neu5Ac derivative, and some of the parainfluenza viruses showed binding to Neu5Gc derivatives containing O-methyl groups. This type of derivative array expanded the diversity of sialic acid compounds that could be analyzed and broadened the knowledge on what types of glycans, in terms of charge, size, and other features, were capable of being bound by well-studied lectins and viruses. Glycoproteins have also served as useful materials to be printed on microarrays (Patwa et al., 2010). While they are inherently heterogeneous compared to defined glycans, they can provide interesting binding data. Some glycoproteins, such as fetuin, have well characterized glycans and so the binding to the glycoprotein can be informative, especially if coupled with treatments such as neuraminidase/sialidase treatments to remove terminal sialic acids.

As mentioned above, many novel sialidases generated by Chen's laboratory were utilized to create sialylated glycan microarrays with interesting modifications to sialic acids including Neu5Gc and 9-O-acetyl derivatives (Yu et al., 2005, 2006a,b), and these arrays were utilized to show the presence of antibodies in patients with carcinomas to a specific glycan, Neu5Gcα2–6GalNAcα1-*O*-Ser/Thr (GcSTn) (Padler-Karavani et al., 2011). Similar arrays were also used to show the novel finding of elevated anti-Gc antibodies in patients with Kawasaki Disease (Padler-Karavani et al., 2013), further demonstrating the usefulness of the array platform. A large-scale study using a sialoglycan microarray generated by the same research groups showed an association of multiple antibodies to Neu5Gc with colorectal cancer risk (Samraj et al., 2018). As described, both the synthetic and natural glycan microarrays have been used to examine a variety of interactions, including those mediated by viral and bacterial proteins, host antibodies, and serum.

The Neoglycolipid (NGL)-based microarray system uses defined glycans, synthetically or naturally obtained, linked to lipids which are then bound to a nitro-cellulose slide (Palma et al., 2014). This platform has been useful in many instances, and was used by Feizi's laboratory to demonstrate that an N-glycolyl GM1-based glycan is a receptor for simian virus 40 (Campanero-Rhodes et al., 2007). They have incorporated various sialic acid-containing glycans into their NGL array platforms, including NeuGc and polysialic acid, and has also made discoveries on binding of other types of samples, such as human adenovirus 52 (Lenman et al., 2018). In addition, N-glycolyl sialic acid was discovered as a binding partner for human polyomavirus 9 viral proteins, further demonstrating the utility of these array formats (Khan et al., 2014).

Bifunctional linkers useful for both the detection of HPLC-separated glycan species and direct printing of glycans to NHS slides have been developed (Song et al., 2009, 2015; Yamada et al., 2013), such as AEAB and Fmoc derivatives, which allow for easy purification and quantification, in addition to the fluorescent properties and printing capabilities. Surface plasmon resonance (SPR) can be used in place of fluorescence as an indicator of binding interactions (de Boer et al., 2008). SPR studies coupled with glycan array studies have subsequently been used to look at sialic acid binding, specifically comparing the ability of proteins to bind to NeuAc vs. NeuGc, and making discoveries on host-pathogen interactions (Atack et al., 2018).

### Limitations of Microarray Approaches

Microarrays have been an incredible tool for investigating binding interactions on a solid surface between the glycans covalently linked to slides and soluble GBPs. However, there are some limitations to the technique. The most straightforward issue is in the number of glycans that are available for addition to the microarrays. There are predicted to be thousands of glycans in the human glycan repertoire (Cummings, 2009; Cummings and Pierce, 2014), however only a fraction of these are accessible as known glycan structures for incorporation into arrays. There is also a restriction in the enzyme availability- there are not enzymes available to generate every known glycan linkage that has been identified. Additionally, some studies indicate that solution-based experiments are advantageous over solid phase. In order to increase accessibility and address the nature of in solution vs. fixed surface interactions, many groups are investigating the generation of glycan arrays on alternative substrates, such as beads, that will allow for flow cytometric analysis (Purohit et al., 2018). Similarly, cell-based arrays allow for a biologically relevant presentation of glycans (Briard et al., 2018).

## Sialic Acid Binding Reagents

There are a number of reagents that are used in the field for identifying sialic acids. These include using plant or animal lectins, which are naturally occurring glycan-binding proteins, as well as anti-glycan antibodies. On the glycan microarrays, these reagents are detected fluorescently, either due to their direct fluorescence, or with a fluorescently labeled secondary reagent such as streptavidin.

Specifically, *Sambucus nigra* agglutinin (SNA), *Maackia amurensis* leukoagglutinin (MAL-I), and *Maackia amurensis* hemagglutinin (MAH or MAL-II) are all lectins that have been well-characterized in their binding motifs, favoring glycans that contain sialic acid. SNA is a bark lectin from the elderberry plant that has a high affinity toward structures containing terminal Neu5Acα2-6Galβ-. It has unique abilities to differentiate and bind favorably to α2-6-linked sialic acid (Sia)-containing ligands over α2-3-linked sialylated glycans (Cummings and Schnaar, 2017). Binding of SNA to the CFG glycan array shows a strong preference to the Ac version of sialic acid over the Gc, with only one of the Gc compounds being bound. MAL-I and MAL-II are both derived from the leguminous tree, *M. amurensis*, but have diverse binding profiles and affinities. MAL-I has consistently shown affinity toward terminal Neu5Acα2-3 residues that are linked to type-2 N-acetyllactosamine sequences, such as Siaα2-3Galβ1-4GlcNAcβ-Man-R. Studies have shown that this lectin does not bind isomers that contain sialic acid in a α2-6 linkage, with strong preference for α2-3 linkages. MAL-I has also shown binding to glycans that are sulfated as opposed to sialylated with the typical sequence, sulfo-3Galβ1-4GlcNAcβ-Man-R (Cummings and Schnaar, 2017). Analyzing data from MAL-I on the CFG array revealed high binding toward gangliosides that have Gc in their structure, either in a α2-3 or α2-8 linkage, in addition to the Ac and negatively-charged sulfate binding. The binding is less influenced by the Gc and Ac versions of sialic acid than SNA. MAL-II has distinct binding to sialylated core 1 O-glycan Siaα2-3Galβ1-3GalNAcα1-Ser/Thr. It does not exhibit binding to the Gc compounds present on the CFG array. The specificity of these lectins tested on other array platforms shows that it is not just the presence of Ac or Gc sialic acid that effects binding, but that the underlying structure is important, and these specificities are described in more detail in the respective publications (Padler-Karavani et al., 2011, 2012; Song et al., 2011b; Wang et al., 2014).

Commercially available antibodies that are specific in recognizing sialic acid are difficult to find, but the companies Biolegend and Lectenz Bio (www.Lectenz.com) have reagents designated for this purpose. These reagents provide the field with more screening tools for biological samples. Lectenz Bio has a reagent that specifically targets α2-3 linked sialo-glycans over α2-6 and α2-8 linked sialo-glycans, which is similar binding specificity to MAL-I. Another anti-glycan reagent produced by Lectenz Bio aims to broadly identify glycans containing sialic acid in general, independent of the linkage. It remains to be seen whether these reagents can discriminate Ac and Gc. The anti-Neu5Gc antibody from Biolegend is particularly important in studies looking at the effects of the intake and incorporation of Neu5Gc in humans, which has been associated with inflammation and worsening of some diseases (Samraj et al., 2017). The anti-Gc antibodies appear to be specific for Gc compounds and not Ac compounds, and these antibodies are the subject of another review in this series (Dhar et al., 2019).

## Comparative Analysis of Glycan Microarrays and Data Output

All aspects of glycan microarray technology have advanced significantly from chemical and enzymatic generation of the glycans, to novel release methods, to the development of more efficient functional linkers and immobilization strategies (Gagarinov et al., 2017). As the field continues to develop, we are able to further refine the assays and find new uses for the existing glycan microarrays, as well as modify the existing structures on both defined and natural arrays to create new epitopes for binding studies. The MAGS approach (Smith and Cummings, 2013) has been used in conjunction with MS data to sequence unknown glycans, however the same process can be viewed from the perspective of characterizing relevant enzymes and lectins, such as defining the acute specificity of bacterial neuraminidases or the binding nuances of common lectins. Additionally, as more data is generated, a comprehensive comparative approach allows for a link to be established between existing glycoproteomics databases and glycan microarray data. The lectin-glycan interaction (LGI) network enables the prediction of host receptor proteins for pathogenic adhesins (Ielasi et al., 2016). The incredible volume of data generated from the various synthetic and natural glycan microarrays will be invaluable as more is discovered about the GBPs and predictive analysis is developed.

One head-to-head comparison of two similar but novel sialic acid derivative arrays was performed, using the same well-characterized lectins and proteins, and a cross-analysis of the slide images was performed to assess variability (Padler-Karavani et al., 2012). The arrays contained many glycans that were structurally identified, and others with subtle differences in comparison. They were also printed on 2 different slide surfaces, which was noted as a major contributing factor to the differences in binding profiles. The data demonstrates that arrays with the same or similar types of compounds can be complementary to one another. Nevertheless, they should also be compared between laboratories with caution, since there are many variables that contribute to data acquisition, including slide surface, printing method and concentration, buffer conditions, and analysis methods (Padler-Karavani et al., 2012), although we and other groups see very consistent results in terms of reproducibility within the same print batches across many types of glycans and linkers. This and other studies have shown that the properties and lengths of linkers used, the immobilization strategy and slide matrix chemistry, and density of immobilized glycans or glycoproteins can all affect recognition and therefore need to be considered in the data analysis and interpretation steps, and variations in these components could lead to differences in binding across different array platforms (Song et al., 2009; Padler-Karavani et al., 2012; Wang et al., 2014; Gildersleeve and Wright, 2016; Gao et al., 2019; Temme et al., 2019).

The MIRAGE efforts were initiated to aid in the cross-comparison of arrays. MIRAGE, or the Minimum Information Required for A Glycomics Experiment, has been setting the standards in the field for performing and reporting on the array methods and data analysis (Kolarich et al., 2013; York et al., 2014; Struwe et al., 2016; Liu et al., 2017; Campbell et al., 2019). These efforts will continue to benefit the collection of consistent array data across different types of arrays and from different research groups.

Another effort that is paving the way for cross-analyses and centralization of array data is the GlyGen program, https://www.glygen.org. This NIH-funded program is a resource for the glycoscience fields, as a data integration and dissemination tool. Data is retrieved from multiple international sources related to glycan microarrays and other Glycomics data as well as information on the proteins that are tested, and through bioinformatics approaches the data is integrated into a common portal to allow detailed searches and exploration of data. These cross-comparison and integration efforts will allow for validation of data obtained from the myriad of sialic acid-containing glycan microarrays that are currently present in the field.

## Concluding Remarks

The platform of glycan microarrays, and specifically arrays containing sialic acid derivatives such as Neu5Ac and Neu5Gc, have greatly accelerated studies of GBPs and their glycan interactors. These efforts will continue to expand and diversify as the significance of glycan recognition for glycoscience in human immunity, cancer therapy, and host-pathogen interactions is better understood and appreciated, and as new tools are introduced to the research community.

## Author Contributions

AM, LB-L, JH-M, and RC contributed to the writing of the review manuscript.

### Conflict of Interest Statement

The authors declare that the research was conducted in the absence of any commercial or financial relationships that could be construed as a potential conflict of interest.
